# Machine learning for predicting the survival in osteosarcoma patients: Analysis based on American and Hebei Province cohort

**DOI:** 10.17305/bb.2023.8804

**Published:** 2023-10-01

**Authors:** Yahui Hao, Di Liang, Shuo Zhang, Siqi Wu, Daojuan Li, Yingying Wang, Miaomiao Shi, Yutong He

**Affiliations:** 1 Cancer Institute, The Fourth Hospital of Hebei Medical University/The Tumor Hospital of Hebei Province, Shijiazhuang, China

**Keywords:** Osteosarcoma, prognosis model, TNM group, Surveillance, Epidemiology, and End Results (SEER), Hebei Province

## Abstract

Osteosarcoma, a rare malignant tumor, has a poor prognosis. This study aimed to find the best prognostic model for osteosarcoma. There were 2912 patients included from the Surveillance, Epidemiology, and End Results (SEER) database and 225 patients from Hebei Province. Patients from the SEER database (2008–2015) were included in the development dataset. Patients from the SEER database (2004–2007) and Hebei Province cohort were included in the external test datasets. The Cox model and three tree-based machine learning algorithms (survival tree [ST], random survival forest [RSF], and gradient boosting machine [GBM]) were used to develop the prognostic models by 10-fold cross-validation with 200 iterations. Additionally, performance of models in the multivariable group was compared with the TNM group. The 3-year and 5-year cancer-specific survival (CSS) were 72.71% and 65.92% in the development dataset, respectively. The predictive ability in the multivariable group was superior to that in the TNM group. The calibration curves and consistency in the multivariable group were superior to those in the TNM group. The Cox and RSF models performed better than the ST and GBM models. A nomogram was constructed to predict the 3-year and 5-year CSS of osteosarcoma patients. The RSF model can be used as a nonparametric alternative to the Cox model. The constructed nomogram based on the Cox model can provide reference for clinicians to formulate specific therapeutic decisions both in America and China.

## Introduction

Osteosarcoma, a rare malignant tumor, accounts for approximately 35% of primary malignant bone and joint tumors [1, 2]. Osteosarcoma often occurs in children and adolescents, invades the metaphysis of appendicular skeleton, and has a poor prognosis [3–5]. Although the 5-year survival rate of osteosarcoma in the United States has increased from 51.0% (in the 1980s) to 60.5% (in the 2010s), it is still the lowest among bone sarcomas [1]. It is an imperative to develop an accurate survival prediction model that influences the decisions of clinicians, patients, and their families [6]. At present, most models for predicting osteosarcoma are Cox proportional hazard regression models combined with nomograms [3, 7–13]. The Cox models need to meet the proportional hazards assumption, thus the overall quality of these models may have not reached the optimal state.

Machine learning is a new type of artificial intelligence, which has been widely used in medical data analysis and is a powerful tool for improving clinical strategies [14–16]. Some tree-based machine learning methods (such as survival tree [ST], random survival forest [RSF], and gradient boosting machine [GBM]) can account for interaction and effect modification between variables and have been applied in some prognosis studies [17–27]. In many studies, in which the categorical variable was the dependent variable, the prediction performance of machine learning is better than that of traditional models [6, 14, 28, 29]. However, there is little research comparing survival prediction models for osteosarcoma, in which the dependent variable includes survival status and survival time. It is unclear whether the performance of the new machine learning model is superior to the traditional Cox regression model for survival prediction.

In this study, we selected patients with osteosarcoma from the National Cancer Institute Surveillance, Epidemiology, and End Results (SEER) database (from 2008 to 2015) to develop four prognosis models (Cox, ST, RSF, and GBM). Patients with osteosarcoma from the SEER database (from 2004 to 2007) and Hebei Province cancer registry in North China were used as external test dataset 1 and external test dataset 2, respectively. In addition, we compared the multivariable group with the TNM group (included only 7th American Joint Committee on Cancer [AJCC] T, N, and M category).

This study aimed to find the optimal survival prediction model for osteosarcoma, in order to help clinicians make more reasonable therapeutic decisions both in America and China.

## Materials and methods

### Data source and study population

Data of SEER cohort were obtained from the “Incidence - SEER Research Plus Data, 17 Registries, Nov2021 Sub (2000–2019)” of the SEER database, a population-based cancer registry of the National Cancer Institute in the United States (https://seer.cancer.gov/). A total of 2912 osteosarcoma patients from the SEER database were included in this study. The inclusion criteria for the patient were the following: (1) Year of diagnosis: “2004–2015”; (2) Site recode ICD-O-3/WHO 2008: “Bones and Joints,” and behavior code ICD-O-3: “Malignant”; (3) AYA site recode 2020 Revision: “Osteosarcoma”; and (4) First malignant primary indicator. The exclusion criteria were the following: (1) Missing survival information (diagnostic confirmation was “autopsy”, “death certificate only cases”, or “unknown”); and (2) Survival months less than one month or unknown.

The Hebei Province cohort was recruited from Hebei Province cancer registry in North China. We included 225 patients who were first diagnosed with osteosarcoma between 1 January 2008 and 31 December 2021. Patients were followed-up by passive and active methods. Passive follow-up information was collected through readmission information, outpatient records, and all cause of death database in Hebei Province. Active follow-up was conducted trimonthly by professionally trained personnel. The end date for follow-up was 20 January 2022. Second, we excluded those with incomplete survival information (missing survival information) or survival months less than one month or unknown.

### Predictors and outcome

We included 11 predictors (sex, age, marital status, site, T category, N category, M category, grade, surgery, radiation, and chemotherapy) into the ST, RSF, and GBM models of the multivariable group. As the Cox proportional hazard model needs to meet the proportional hazards assumption, Cox model included only the variables without overlapping in Kaplan–Meier curves and those having statistically significant difference in log-rank test. In addition, we compared the multivariable group with the TNM group (only included 7th AJCC T category, N category, and M category). To avoid bias caused by different variables, we set the same variables group (four models included only variables that met the proportional hazards assumption). Survival months were calculated from the date of diagnosis to the date of death due to osteosarcoma or the end of follow-up. The outcome variables were survival months and 3-year and 5-year cancer-specific survival (CSS).

### Model development and testing

In this study, patients in the SEER cohort from 2008 to 2015 were assigned to the development dataset. Patients from 2004 to 2007 comprised the external test dataset 1. Patients from Hebei Province cohort formed the external test dataset 2. Cox, ST, RSF, and GBM were used to develop the prediction models. All models were built on the development dataset by 10-fold cross-validation with 200 iterations. Discrimination of the survival prediction models was quantified by concordance index (C-index) and area under the receiver operating characteristic curve (AUC). Calibration curves were used to evaluate the consistency between the predicted and observed values of CSS of the survival prediction models.

Cox is a popular semi-parametric model for survival analysis, which can be defined by formula:

$$h(t)=h_{0}(t)e^{\beta_{1}X_{1}+\beta_{2}X_{2}+\cdots +\beta_{p}X_{p}}$$

where *t* represents the survival time and β_*p*_ is the coefficient of covariate *X*_*p*_ and measures the impact of the covariate. *h*(*t*) is the hazard function of individuals with covariates *X*_1_, *X*_2_,…,*X*_*p*_ at time *t*. *h*_0_(*t*) is the baseline hazard.

The CART algorithm was used to construct the tree structure [30, 31]. The procedure consisted of three steps. First, we examined all allowable splits on each predictor variable and choose a split point that maximizes the survival differences between children nodes based on the log-rank test. Second, we repeated the procedure to split the children nodes until the tree met the stopping criteria (all terminal nodes containing only the minimum number of unique events). Third, the split-complexity measure was used in the pruning step.

RSF is an ensemble tree method for the analysis of right-censored survival data [32]. The algorithm of RSF was the following: First, B bootstrap samples were drawn from the original data. Each bootstrap sample, including two-thirds of the original data, was used to train data. Second, in order to grow a ST for each bootstrap sample, we randomly selected *p* candidate variables at each node of the tree. The criterion of growing a tree was the maximization of the survival difference between each branch. Third, the cumulative hazard function (CHF) of each tree was averaged to achieve an ensemble CHF. Finally, the prediction error for the ensemble CHF was calculated, by using out-of-bag data (OOB, the rest one-third of the original data) to avoid overfitting.

GBM is a gradient-descent-based formulation of boosting methods [33]. The basic idea is to train new base learners according to the negative gradient information of the loss function of the current model. Then, it combines the trained base learners with the existing model in the form of accumulation. This process aimed to continuously reduce the loss function and deviation.

### Ethical statement

The authors are accountable for all aspects of the work in ensuring that questions related to the accuracy or integrity of any part of the work are appropriately investigated and resolved. The study was conducted in accordance with the Declaration of Helsinki (as revised in 2013). The authors obtained authorization to access the SEER Research Data supported by the National Cancer Institute with approval number 11241-Nov2021. Because public and anonymous data from the SEER database were used, informed patient consent was not required. The Ethics Committee of the Fourth Hospital of Hebei Medical University/The Tumor Hospital of Hebei Province has confirmed that no ethical approval was required. Informed consent was obtained from all individual participants included in the study.

### Statistical analysis

The analyses were conducted using the statistical software R (version 4.1.2). R package “randomForestSRC” was used for missing data imputation. R packages “survival,” “rpart,” “randomForestSRC,” and “gbm” were used to develop the models. The value of *P* < 0.05 was considered statistically significant.

## Results

### Demographic characteristics of patients

**Figure 1.**
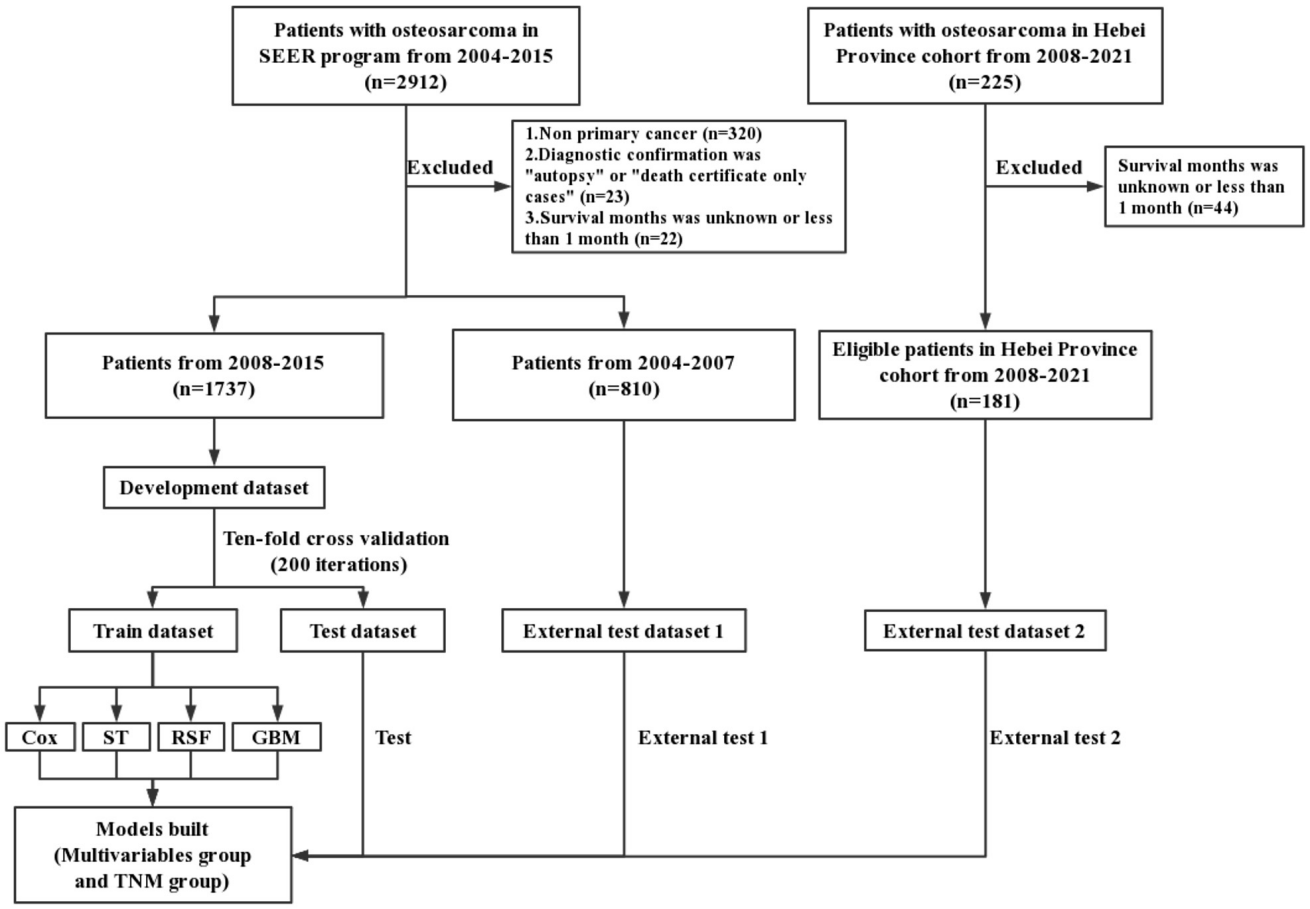
**Flowchart of study design and patient selection.** SEER: Surveillance, Epidemiology, and End Results; ST: Survival tree; RSF: Random survival forest; GBM: Gradient boosting machine; TNM: Tumor, node, metastasis.

Figure 1 depicts the flowchart of study design and patient selection. A total of 2912 patients with osteosarcoma were diagnosed from 2004 to 2015 in the SEER program. We excluded 365 patients who did not meet the inclusion criteria. Finally, 1737 patients (from 2008 to 2015) were included in the development dataset and 810 patients (from 2004 to 2007) were included in the external test dataset 1. The Hebei Province cohort included 225 patients. Finally, 181 patients were included in the external test dataset 2 in accordance with the inclusion and exclusion criteria. The 3-year and 5-year CSS were 72.71% and 65.92% in the development dataset, 70.25% and 63.46% in the external test dataset 1, and 55.25% and 52.49% in the external test dataset 2, respectively (Figure 1, Table 1, and Table S1).

**Table 1:** Demographic characteristics of patients with osteosarcoma in imputation data

	**Variables**	**Development dataset**	**External test dataset 1**		**External test dataset 2**	
		***N* (%)**	***N* (%)**	***P* value^a^**	**N (%)**	***P* value^b^**
Total		1737 (100)	810 (100)		181 (100)	
Sex				0.298		0.363
	Male	954 (54.9)	427 (52.7)		93 (51.4)	
	Female	783 (45.1)	383 (47.3)		88 (48.6)	
Age (years)				0.608		<0.001
	0–19	921 (53.0)	441 (54.4)		53 (29.3)	
	20–39	415 (23.9)	174 (21.5)		45 (24.9)	
	40–59	246 (14.2)	119 (14.7)		50 (27.6)	
	≥60	155 (8.9)	76 (9.4)		33 (18.2)	
Marital status				0.220		<0.001
	Married	379 (21.8)	180 (22.2)		104 (57.5)	
	Unmarried	1280 (73.7)	581 (71.7)		75 (41.4)	
	Other^c^	78 (4.5)	49 (6.0)		2 (1.1)	
Site				0.587		<0.001
	Appendicular skeleton	1573 (90.6)	728 (89.9)		101 (55.8)	
	Pelvis and spine	164 (9.4)	82 (10.1)		80 (44.2)	
T category				0.070		<0.001
	T1	702 (40.4)	365 (45.1)		78 (43.1)	
	T2	981 (56.5)	418 (51.6)		89 (49.2)	
	T3	54 (3.1)	27 (3.3)		14 (7.7)	
N category				0.762		<0.001
	N0	1695 (97.6)	792 (97.8)		162 (89.5)	
	N1	42 (2.4)	18 (2.2)		19 (10.5)	
M category				0.394		0.018
	M0	1409 (81.1)	658 (81.2)		151 (83.4)	
	M1a	188 (10.8)	77 (9.5)		9 (5.0)	
	M1b	140 (8.1)	75 (9.3)		21 (11.6)	
Grade				0.898		0.819
	Well differentiated or moderately differentiated	173 (10.0)	82 (10.1)		19 (10.5)	
	Poorly differentiated or undifferentiated	1564 (90.0)	728 (89.9)		162 (89.5)	
Surgery				0.280		0.056
	No	236 (13.6)	123 (15.2)		34 (18.8)	
	Yes	1501 (86.4)	687 (84.8)		147 (81.2)	
Radiation				0.488		<0.001
	No	1617 (93.1)	760 (93.8)		143 (79.0)	
	Yes	120 (6.9)	50 (6.2)		38 (21.0)	
Chemotherapy				0.542		<0.001
	No	346 (19.9)	153 (18.9)		75 (41.4)	
	Yes	1391 (80.1)	657 (81.1)		106 (58.6)	

^a^*P* value was derived from the chi-square test comparing the development dataset and external test dataset 1; ^b^*P* value was derived from the chi-square test comparing the development dataset and external test dataset 2; ^c^Others include divorced, separated, and widowed.

Of the 1737 patients in the development dataset, 954 (54.9%) were male and 783 (45.1%) were female. There were 921 (53.0%), 415 (23.9%), 246 (14.2%), and 155 (8.9%) patients who were 0–19 years, 20–39 years, 40–59 years, and ≥ 60 years old, respectively. Regarding marital status, 379 (21.8%) were married and 1280 (73.7%) were not married. Osteosarcoma occurred in appendicular skeleton (including long and short bones of the upper and lower extremities) in 1573 (90.6%) patients and occurred in pelvis, spine and skull in 164 (9.4%) patients. Regarding the T category, 702 (40.4%) were T1, 981 (56.5%) were T2, and 54 (3.1%) were T3. Only 42 (2.4%) were N1. Regarding the M category, 1409 (81.1%) were M0, 188 (10.8%) were M1a, and 140 (8.1%) were M1b. Regarding grade, 173 (10.0%) were well differentiated or moderately differentiated, whereas 1564 (90.0%) were poorly differentiated or undifferentiated. Regarding treatment, 1501 (86.4%) patients underwent surgery, 120 (6.9%) received radiation therapy, and 1391 (80.1%) received chemotherapy. In the imputation data, the detailed characteristics of patients with osteosarcoma in the development dataset, external test 1, and external test 2 are shown in Table 1.

### Model performance

**Figure 2.**
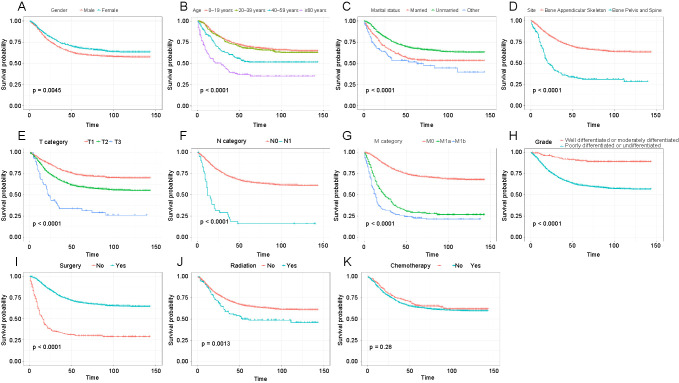
**Kaplan–Meier curves for different variables:** (A) Sex; (B) age; (C) marital status; (D) tumor sites; (E) T categories; (F) N categories; (G) M categories; (H) grades; (I) surgery; (J) radiation; (K) chemotherapy.

According to the Kaplan–Meier curves and log-rank tests for different variables (Figure 2), the variables which met the proportional hazards assumption were sex, age, marital status, site, T category, N category, M category, grade, surgery, and radiation. Those variables were included in the Cox model of the multivariable group. As the ST, RSF, and GBM models did not need to meet the proportional hazards assumption, the included variables of the multivariable group were sex, age, marital status, site, T category, N category, M category, grade, surgery, radiation, and chemotherapy.

**Figure 3.**
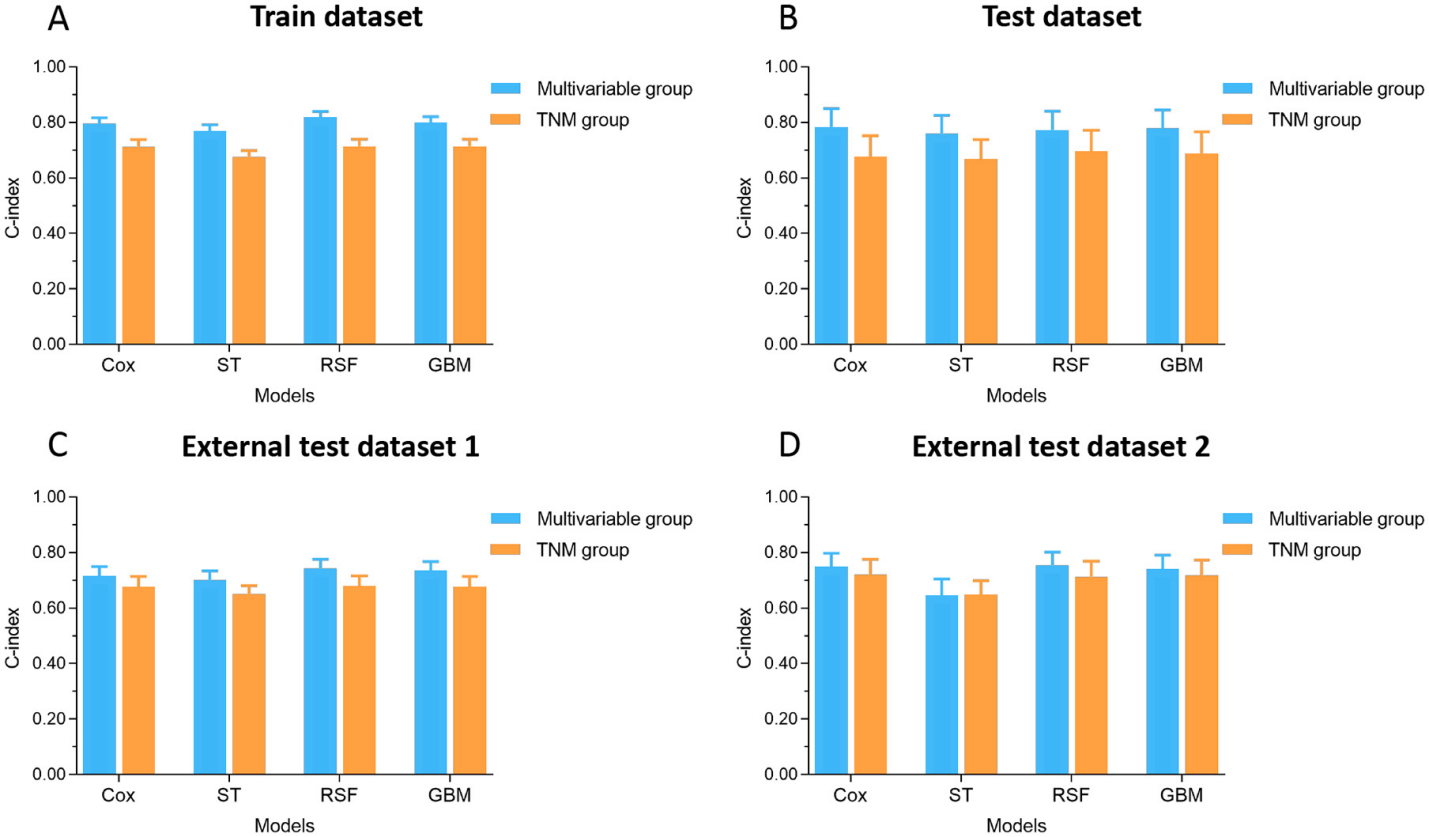
**Histogram of C-indices for various models for predicting 3-year CSS of osteosarcoma in the multivariable group and TNM group:** (A) Train dataset; (B) Test dataset; (C) External test dataset 1; (D) External test dataset 2. CSS: Cancer-specific survival. C-index: Concordance index; SEER: Surveillance, Epidemiology, and End Results; ST: Survival tree; RSF: Random survival forest; GBM: Gradient boosting machine; TNM: Tumor, node, metastasis.

To compare the performance of the four models (Cox, ST, RSF, and GBM), we computed the C-index and plotted the receiver operating characteristic curves (ROC) and calibration curves in 3-year survival cohort and 5-year survival cohort of different datasets. Figure 3 and Table 2 show the C-indices for various models for predicting 3-year CSS of osteosarcoma in the multivariable group and TNM group. The C-indices in train datasets of the multivariable group were 0.80 (0.75–0.84), 0.77 (0.73–0.81), 0.82 (0.78–0.86), and 0.80 (0.76–0.84) for the Cox, ST, RSF, and GBM models, respectively. In the multivariable group, except for ST in the external test dataset 2, the C-indices of other models were higher than 0.70. The C-indices in train datasets of the TNM group were 0.71 (0.67–0.75), 0.68 (0.64–0.72), 0.71 (0.65–0.77), and 0.71 (0.65–0.77) for the Cox, ST, RSF, and GBM models, respectively. In the TNM group, the C-indices of the Cox, RSF, and GBM models were higher than 0.70 in the train dataset and external test dataset 2. The C-indices for various models of every dataset in the multivariable group were higher than that in the TNM group. The C-indices of other datasets or models were not higher than 0.70. The C-indices of the Cox, RSF, and GBM models were higher than that of the ST model in each dataset both in the multivariable group and in TNM group. Figure S1 and Table S2 show the C-indices for various models for predicting 5-year CSS of osteosarcoma in the multivariable group and TNM group, which had the similar results as for the 3-year CSS cohort.

**Table 2:** C-indices for various models for predicting 3-year CSS of osteosarcoma in the multivariable group and TNM group

**Models**	**Train dataset Mean (95% CI)**	**Test dataset Mean (95% CI)**	**External test dataset 1 Mean (95% CI)**	**External test dataset 2 Mean (95% CI)**
*Multivariable group*				
Cox	0.80 (0.75–0.84)	0.78 (0.65–0.91)	0.72 (0.65–0.78)	0.75 (0.65–0.85)
ST	0.77 (0.73–0.81)	0.76 (0.63–0.89)	0.70 (0.64–0.77)	0.65 (0.53–0.77)
RSF	0.82 (0.78–0.86)	0.77 (0.64–0.90)	0.74 (0.68–0.81)	0.75 (0.65–0.85)
GBM	0.80 (0.76–0.84)	0.78 (0.65–0.91)	0.74 (0.67–0.80)	0.74 (0.64–0.84)
*TNM group*				
Cox	0.71 (0.67–0.75)	0.68 (0.53–0.82)	0.68 (0.60–0.76)	0.72 (0.60–0.84)
ST	0.68 (0.64–0.72)	0.67 (0.53–0.80)	0.65 (0.59–0.71)	0.65 (0.55–0.75)
RSF	0.71 (0.65–0.77)	0.70 (0.55–0.84)	0.68 (0.60–0.76)	0.71 (0.59–0.83)
GBM	0.71 (0.65–0.77)	0.69 (0.54–0.84)	0.68 (0.60–0.76)	0.72 (0.60–0.84)

C-index: Concordance index; CSS: Cancer-specific survival; ST: Survival tree; RSF: Random survival forest; GBM: Gradient boosting machine; TNM: Tumor, node, metastasis.

**Figure 4.**
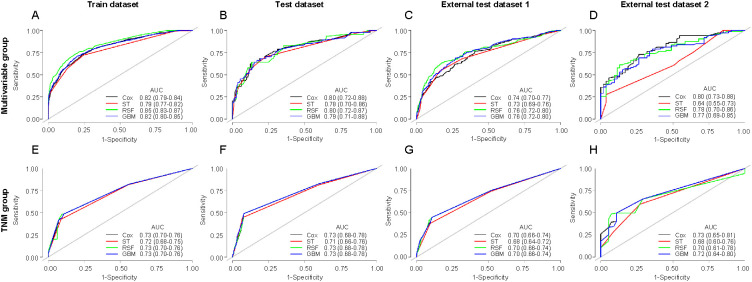
**ROC for various models for predicting 3-year CSS of osteosarcoma in the multivariable group and TNM group:** (A) Train dataset in the multivariable group; (B) Test dataset in the multivariable group; (C) External test dataset 1 in the multivariable group; (D) External test dataset 2 in the multivariable group; (E) Train dataset in the TNM group; (F) Test dataset in the TNM group; (G) External test dataset 1 in the TNM group; (H) External test dataset 2 in the TNM group. ROC: Receiver operating characteristic curve; CSS: Cancer-specific survival; ST: Survival tree; RSF: Random survival forest; GBM: Gradient boosting machine; TNM: Tumor, node, metastasis; AUC: Area under the curve.

Figure 4 shows AUC by picturing ROC for various models for predicting 3-year CSS of osteosarcoma in the multivariable group and TNM group. The AUCs in train datasets of the multivariable group were 0.82 (0.79–0.84), 0.79 (0.77–0.82), 0.85 (0.83–0.87), and 0.82 (0.80–0.85) for the Cox, ST, RSF, and GBM models, respectively. The AUCs in train datasets of the TNM group were 0.73 (0.70–0.76), 0.72 (0.68–0.75), 0.73 (0.70–0.76), and 0.73 (0.70–0.76) for the Cox, ST, RSF, and GBM models, respectively. Except ST in the external test dataset 2, the AUCs of multivariable group were higher than of the TNM group in every model of different datasets. In every dataset, the AUC of the ST model was lower than that of the other three models. Figure S2 shows AUC by picturing ROC for various models for predicting 5-year CSS of osteosarcoma in the multivariable group and TNM group. The results of the 5-year CSS cohort were similar to those of the 3-year CSS cohort.

**Figure 5.**
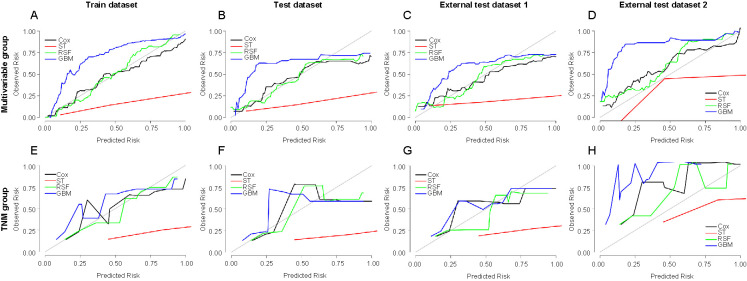
**Calibration curves for various models for predicting 3-year CSS of osteosarcoma in the multivariable group and TNM group:** (A) Train dataset in the multivariable group; (B) Test dataset in the multivariable group; (C) External test dataset 1 in the multivariable group; (D) External test dataset 2 in the multivariable group; (E) Train dataset in the TNM group; (F) Test dataset in the TNM group; (G) External test dataset 1 in the TNM group; (H) External test dataset 2 in the TNM group. CSS: Cancer-specific survival; ST: Survival tree; RSF: Random survival forest; GBM: Gradient boosting machine; TNM: Tumor, node, metastasis.

Calibration curve was used to visualize the consistency between the predicted and observed values of CSS of the survival prediction models. The 45-degree gray straight line represents the perfect match between the observed (*y*-axis) and predicted (*x*-axis) survival probabilities. A smaller distance between model and gray straight line indicates higher accuracy. Figure 5 shows the calibration curves for various models for predicting 3-year CSS of osteosarcoma in the multivariable group and TNM group. In every dataset, the consistency of the multivariable group was superior to the TNM group. In the multivariable group, the consistency of the Cox and RSF models was superior to that of the ST and GBM models. The predicted value of the GBM model was lower than the observed value, and the predicted value of the ST model was higher than the actual value. Figure S3 depicts the calibration curves for various models for predicting 5-year CSS of osteosarcoma in the multivariable group and TNM group. The results of the 5-year CSS cohort were similar to those of the 3-year CSS cohort.

### Outcome of the Cox model

**Figure 6.**
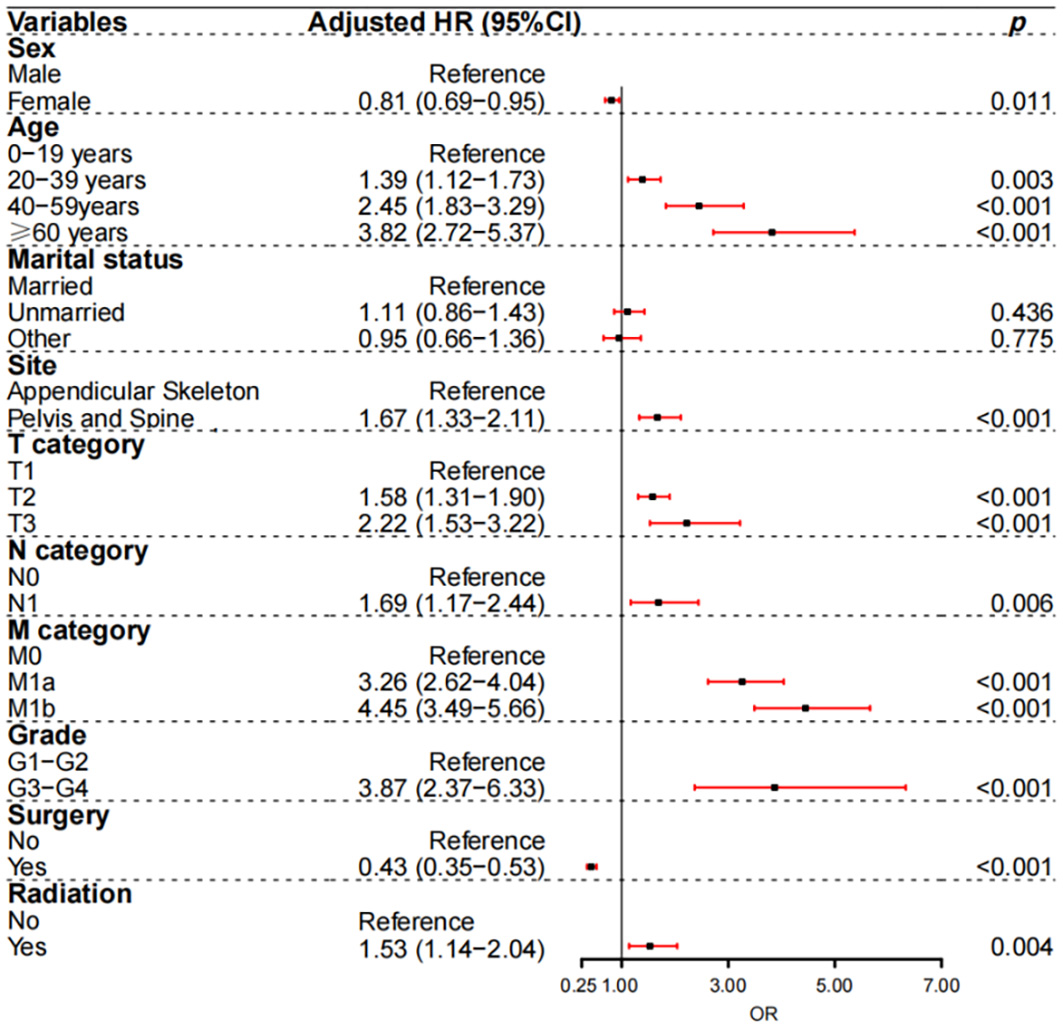
**Influencing factors of osteosarcoma patients prognosis based on the Cox proportional hazard model in development dataset of multivariable group.** G1–G2: Well differentiated or moderately differentiated; G3–G4: Poorly differentiated or undifferentiated.

Figure 6 depicts the factors influencing the prognosis of osteosarcoma patients based on the Cox proportional hazard model in the development dataset of the multivariable group. According to the forest plot, poorer CSS was associated with older age (20–39 years with hazard ratio [HR] 1.39, 95% confidence interval [CI] 1.12–1.73, *P* ═ 0.003; 40–59 years with HR 2.45, 95% CI 1.83–3.29, *P* < 0.001; ≥ 60 years with HR 3.82, 95% CI 2.72–5.37, *P* < 0.001) compared with 0–19 years; having a pelvis and spine tumor site (HR 1.67, 95% CI 1.33–2.11, *P* < 0.001) compared with having an appendicular skeleton tumor site; T2 (HR 1.58, 95% CI 1.31–1.90, *P* < 0.001) or T3 (HR 2.22, 95% CI 1.53–3.22, *P* < 0.001) compared with T1; N1 (HR 1.69, 95% CI 1.17–2.44, *P* ═ 0.006) compared with N0; M1a (HR 3.26, 95% CI 2.62–4.04, *P* < 0.001) or M1b (HR 4.45, 95% CI 3.49–5.66, *P* < 0.001) compared with M0; poorly differentiated or undifferentiated (HR 3.87, 95% CI 2.37–6.33, *P* < 0.001) compared with well differentiated or moderately differentiated; and radiation (HR 1.53, 95% CI 1.14–2.04, *P* ═ 0.004) compared with no radiation. Improved CSS was associated with female sex (HR 0.81, 95% CI 0.69–0.95, *P* ═ 0.011) compared with male sex; and surgery (HR ═ 0.43, 95% CI 0.35–0.53, *P* < 0.001) compared with no surgery.

**Figure 7.**
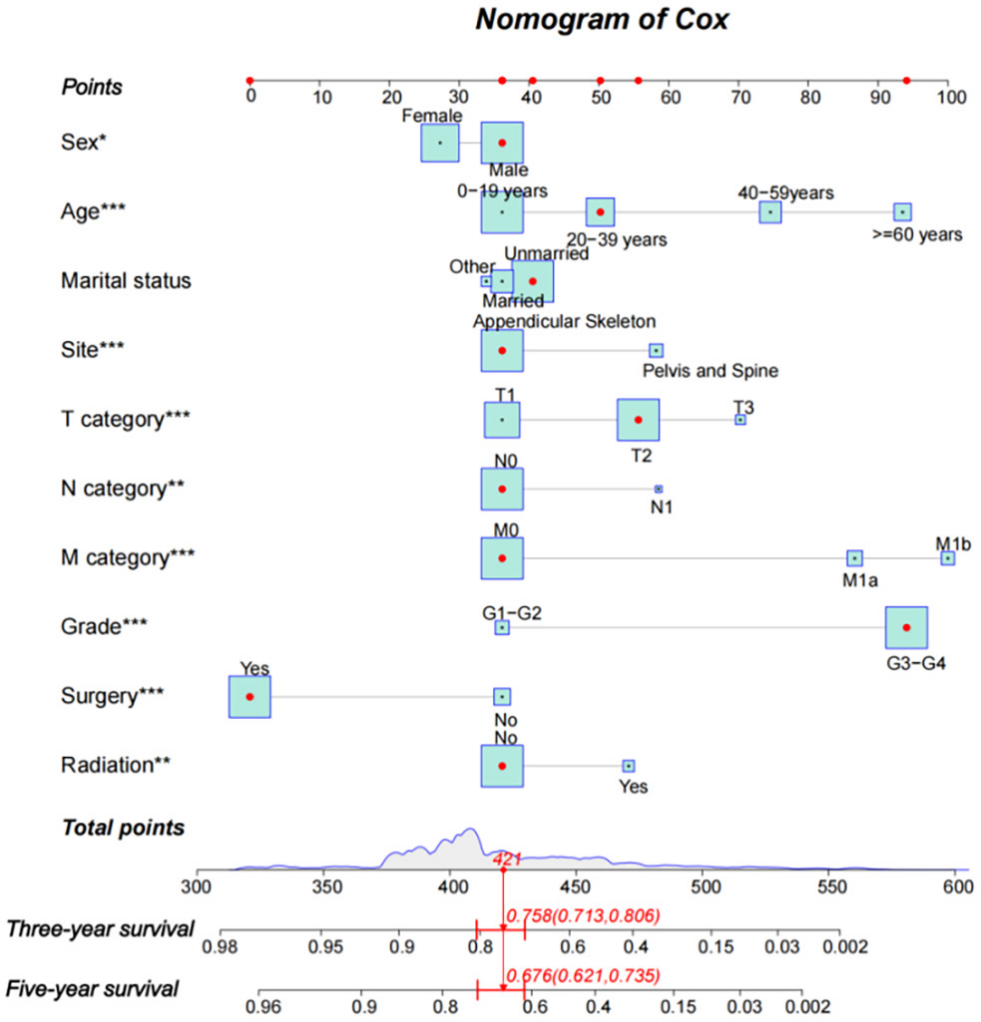
**Nomogram for prediction of the 3-year and 5-year CSS of osteosarcoma patients based on the Cox proportional hazard model in development dataset of the multivariable group.** G1–G2: Well differentiated or moderately differentiated; G3–G4: Poorly differentiated or undifferentiated. CSS: Cancer-specific survival.

Based on the result of the Cox model in the development dataset of the multivariable group, a nomogram was established to predict the 3-year and 5-year CSS of osteosarcoma patients. By bringing the patient’s variable into the nomogram, the scores of each variable can be obtained and, finally, the scores can be added to obtain the patient’s 3-year and 5-year CSS. For example, we included each variable of the patient ID 771469 in the external test set 2 into the nomogram and obtained the score of 412; the 3-year and 5-year CSS of this patient were 0.758 (0.713–0.806) and 0.676 (0.621–0.735), respectively (Figure 7).

### Sensitivity analysis

To avoid bias caused by different variables, we set the same variables group. In the group, the included variables in the ST, RSF, and GBM models were same as in the Cox model, which included sex, age, marital status, site, T category, N category, M category, grade, surgery, and radiation. The C-index, AUC, and calibration curves of same variables group were similar to the multivariable group. (Table S3 and Figures S4 and S5)

## Discussion

Machine learning can improve the efficiency of survival prediction models, which is crucial for disease prognosis and nursing plan. This study developed and validated four models using the Cox and three machine learning algorithms (ST, RSF, and GBM) to predict the survival of osteosarcoma patients. In every model of different dataset, the predictive ability of the multivariable group was superior to the TNM group. In every dataset, the C-index and AUC of the ST were lower than that of the other three models. The calibration curves of the Cox and RSF models performed better than that of the ST and GBM. In addition, the nomogram was established to predict the 3-year and 5-year CSS of osteosarcoma patients.

Previous studies [34–36] showed that there was no difference in the prognosis of osteosarcoma among different ethnic groups. Therefore, it is feasible for us to choose the Hebei Province cohort as the external test dataset 2. Compared with American cohorts (the 3-year and 5-year CSS were 72.71% and 65.92% in the development data, and 70.25% and 63.46% in the external test dataset 1, respectively), the Hebei Province cohort (the 3-year and 5-year CSS were 55.25% and 52.49% in the external test dataset 2, respectively) had a poorer survival. This may be due to the fact that the proportion of pelvis and pine osteosarcoma patients in the Hebei Province cohort (44.2%) is much higher than that in the United States (9.4%). Previous studies [35] have shown that the prognosis of pelvis and pine osteosarcoma patients is worse than that of appendicular skeleton osteosarcoma patients, which is consistent with the Cox regression model in this study.

The C-index and AUC of the Cox model were similar to that of the RSF and GBM models for predicting the survival of osteosarcoma patients. This may be due to the small number of predictors. Cox is a semi-parametric model, which is valid only when the number of predictors is less than the number of events. The advantages of machine learning are more easily seen with large number of predictors and a relatively small sample size [37]. ST model performed worst, in terms of C-index, AUC, or calibration curve. ST divides data by maximizing the difference between nodes, but the prediction error is large, leading to low prediction accuracy of the model. RSF and GBM models can compensate for the error [37]. The performance of the GBM model is similar to the Cox and RSF models for C-index and AUC, but is poor for calibration curve. RSF model can be used as a non-parametric alternative to the Cox model. The selection of methods should be based on a combination of many factors, such as the type of data collected, data size, calculation strength, model implementation skills, and software availability [38].

The Kaplan–Meier curves showed that there were differences in survival between different marital statuses. However, the results of multivariate Cox analysis showed that marital status is not a factor affecting survival. The possible reason is that the proportion of young osteosarcoma patients is higher, and the survival rate of young patients is higher than that of middle-aged and elderly patients. Most young patients are unmarried. This may be the reason why was the survival of unmarried patients higher than that of married patients in the Kaplan–Meier curve. According to the Cox proportional hazard model, poorer CSS was associated with older age compared with 0–19 age group; having a pelvis and spine tumor site compared with having a appendicular skeleton tumor site; T2 or T3 compared with T1; N1 compared with N0; M1a or M1b compared with M0; poorly differentiated or undifferentiated compared with well differentiated or moderately differentiated; and radiation compared with no radiation. Improved CSS was associated with female sex compared with male sex and surgery compared with no surgery. The influencing factors are consistent with previous studies [7, 34, 37, 39–41].

The advantages of this study mainly include the following aspects. First, in order to verify the reliability of the model, all models were built on the development dataset by 10-fold cross-validation with 200 iterations. Previous studies established nomograms based on the Cox regression models using data from a country or region and that have not been validated in the external test dataset [3, 6–8, 42–44]. Our model was selected as the optimal model after comparing various models. In addition, we used not only data in the SEER dataset of 2004–2007 as time cohort external test dataset, but also used Chinese data as regional cohort external test dataset. The external test demonstrated the reliability of our model in different time periods and regions. The nomogram based on the Cox model has a wider application; it is applicable not only to osteosarcoma patients in the United States but also appropriate for Chinese population. Second, to our knowledge, this is the first study to compare multivariable groups of multiple models with the TNM groups for osteosarcoma. Gao et al. [42] established a nomogram compared with AJCC stage to predict the overall survival for postoperative osteosarcoma patients, which was limited to patients with osteosarcoma after surgery and only combined the Cox model and nomogram. In our study, we used the TNM group consisting of three variables (T category, N category, and M category) which is more detailed than the AJCC stage. Third, we did a sensitivity analysis by setting same variables group. In the group, the included variables of the ST, RSF, and GBM models were same as in the Cox model. It had a similar result to multivariable group, which avoided the bias caused by different variables in different models.

There are still some limitations in this study. First, the variables included in this study are limited. There are some studies that demonstrated that some variables (such as gene-based signature, radiomics, pathological fracture, etc.) could also influence the survival in osteosarcoma [43–48]. In future research, we will try to incorporate these factors into the models. Second, the consistency of calibration curves was not high, which may be due to the insufficient sample size of osteosarcoma as a rare cancer. Efforts will be made to solve the problem of low consistency. Third, we included only three machine learning methods to compare with the Cox model. Currently, there are some machine learning or deep learning models based on biomedical images that have been applied in the diagosis of osteosarcoma [46–48]. In the future, we will explore whether these models and biomedical image variables can be used to develop survival prognostic models for osteosarcoma. In addition, we will try to compare more models (such as support vector machine, artificial neural network, Xgboost, deep learning, etc.) to develop a more comprehensive and better prediction model.

## Conclusion

The multivariable group was superior to the TNM group. Among the four prognosis models, the Cox and RSF models performed better than the ST and GBM models. RSF model can be used as a non-parametric alternative to the Cox model. The nomogram based on the Cox model in development dataset of the multivariable group can provide reference for clinicians to formulate specific therapeutic decisions and allocate health resources reasonably for osteosarcoma patients both in America and in China.

## Supplemental Data

Supplementary data can be found at the following link: https://www.bjbms.org/ojs/index.php/bjbms/article/view/8804/2745.
